# Three Rings to Rule Them All: How Versatile Flavoenzymes Orchestrate the Structural Diversification of Natural Products

**DOI:** 10.1021/acs.biochem.1c00763

**Published:** 2021-12-28

**Authors:** Marina Toplak, Robin Teufel

**Affiliations:** Faculty of Biology, University of Freiburg, Schänzlestrasse 1, 79104 Freiburg, Germany

## Abstract

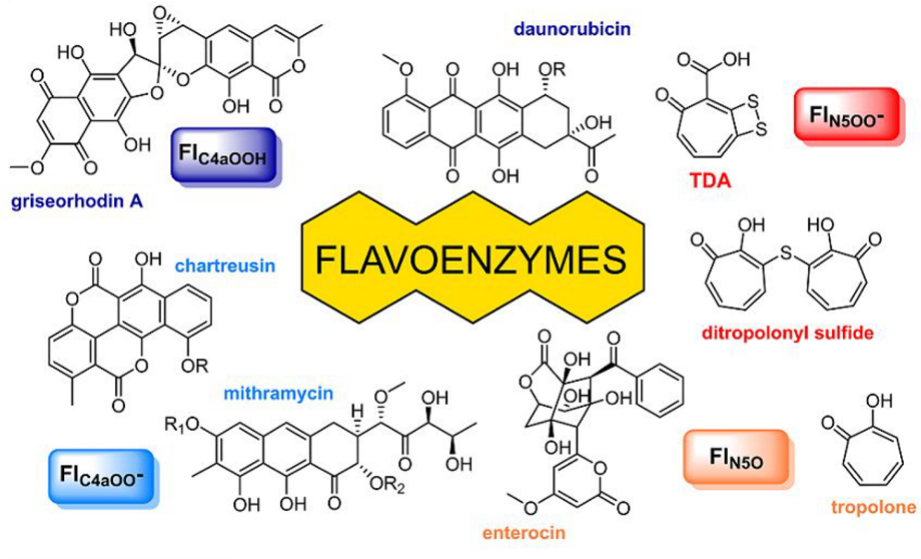

The structural diversification of natural products is instrumental to their versatile bioactivities. In this context, redox tailoring enzymes are commonly involved in the modification and functionalization of advanced pathway intermediates en route to the mature natural products. In recent years, flavoprotein monooxygenases have been shown to mediate numerous redox tailoring reactions that include not only (aromatic) hydroxylation, Baeyer–Villiger oxidation, or epoxidation reactions but also oxygenations that are coupled to extensive remodeling of the carbon backbone, which are often central to the installment of the respective pharmacophores. In this Perspective, we will highlight recent developments and discoveries in the field of flavoenzyme catalysis in bacterial natural product biosynthesis and illustrate how the flavin cofactor can be fine-tuned to enable chemo-, regio-, and stereospecific oxygenations via distinct flavin-C4a-peroxide and flavin-N5-(per)oxide species. Open questions remain, e.g., regarding the breadth of chemical reactions enabled particularly by the newly discovered flavin-N5-oxygen adducts and the role of the protein environment in steering such cascade-like reactions. Outstanding cases involving different flavin oxygenating species will be exemplified by the tailoring of bacterial aromatic polyketides, including enterocin, rubromycins, rishirilides, mithramycin, anthracyclins, chartreusin, jadomycin, and xantholipin. In addition, the biosynthesis of tropone natural products, including tropolone and tropodithietic acid, will be presented, which features a recently described prototypical flavoprotein dioxygenase that may combine flavin-N5-peroxide and flavin-N5-oxide chemistry. Finally, structural and mechanistic features of selected enzymes will be discussed as well as hurdles for their application in the formation of natural product derivatives via bioengineering.

Natural products (i.e., secondary metabolites) are structurally diverse and mostly generated by microorganisms (bacteria and fungi) and plants. It is assumed that they increase the survivability of the producing organism in its natural environment.^1−3^ The biosynthesis of the natural products often starts from simple activated building blocks and involves characteristic core enzymes depending on the compound class. Tailoring enzymes are then responsible for the structural diversification and functionalization of advanced intermediates and are therefore essential for enabling specific interactions of the mature natural products with their molecular targets. In this regard, redox tailoring enzymes and particularly oxygenases such as cytochrome P450 enzymes^4,5^ or flavoenzymes [dependent on flavin adenine dinucleotide (FAD) or flavin mononucleotide (FMN)]^6−9^ adopt important roles. While some catalyze conventional hydroxylation or epoxidation reactions, others couple such oxygen transfer reactions to complex backbone rearrangements that occasionally involve the cleavage and/or formation of multiple carbon–carbon or carbon–heteroatom bonds. As such, they are considered key players for the formation of numerous intricate pharmacophores, which are pivotal for the bioactivity of the mature natural products.^6,10,11^ Studying the structural and mechanistic features of these enzymes is therefore fundamental to gain an understanding of how molecular complexity is generated in nature. Such knowledge also poses a prerequisite for bioengineering efforts aimed at generating novel natural product derivatives, e.g., by rational enzyme design. In this Perspective, we briefly highlight recent discoveries of noncanonical bacterial flavoprotein oxygenases in the biosynthesis of aromatic polyketides that are produced by type II polyketide synthases in conjunction with tailoring enzymes (Figure 1a,b).^12^ In addition, the role of flavoenzymes in the biosynthesis of bacterial tropone natural products, which are produced via an unusual pathway that comprises enzymes from primary and secondary metabolism, will be discussed (Figure 1c).^3^ In this regard, we will also illustrate the chemical properties and reactivities of the distinct oxygenating species produced by the involved flavoenzymes. Finally, we will summarize current open questions and challenges in the field with regard to the identification of novel reactions and underlying enzyme mechanisms as well as the application of these enzymes for the engineering of “unnatural” natural products.

**Figure 1:**
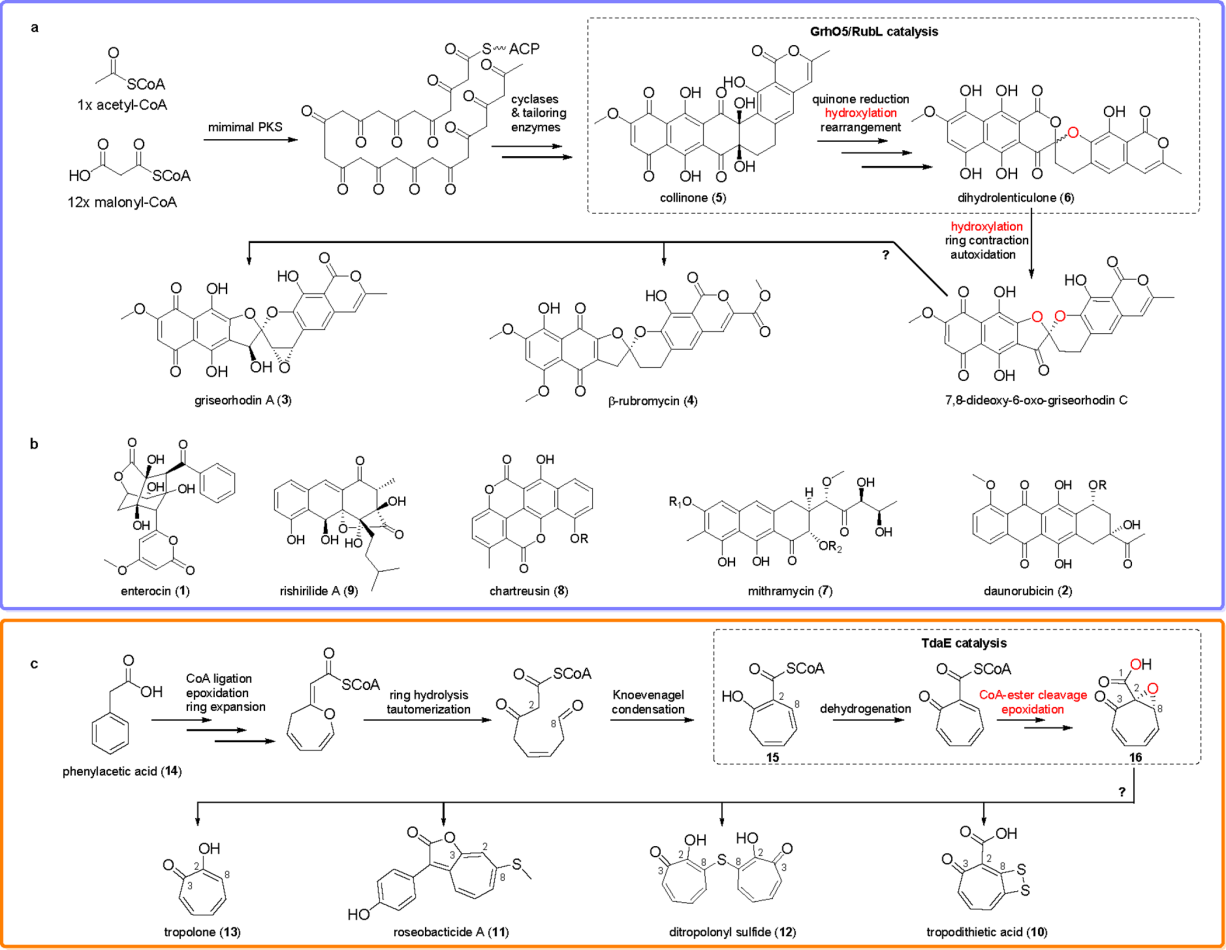
Simplified overview of the bacterial biosynthesis of rubromycin-type polyketides (panel a) as well as other examples of mature aromatic polyketides (panel b) produced by type II polyketide synthases (PKS). The biosynthesis of tropones is shown in panel c. Key tailoring reactions catalyzed by GrhO5/RubL and TdaE are highlighted with dashed boxes. See the text for details. O_2_-derived oxygen atoms incorporated by flavoprotein mono- and dioxygenases are colored red.

## Flavin Oxygenating Species in Natural Product Biosynthesis

**Figure 2:**
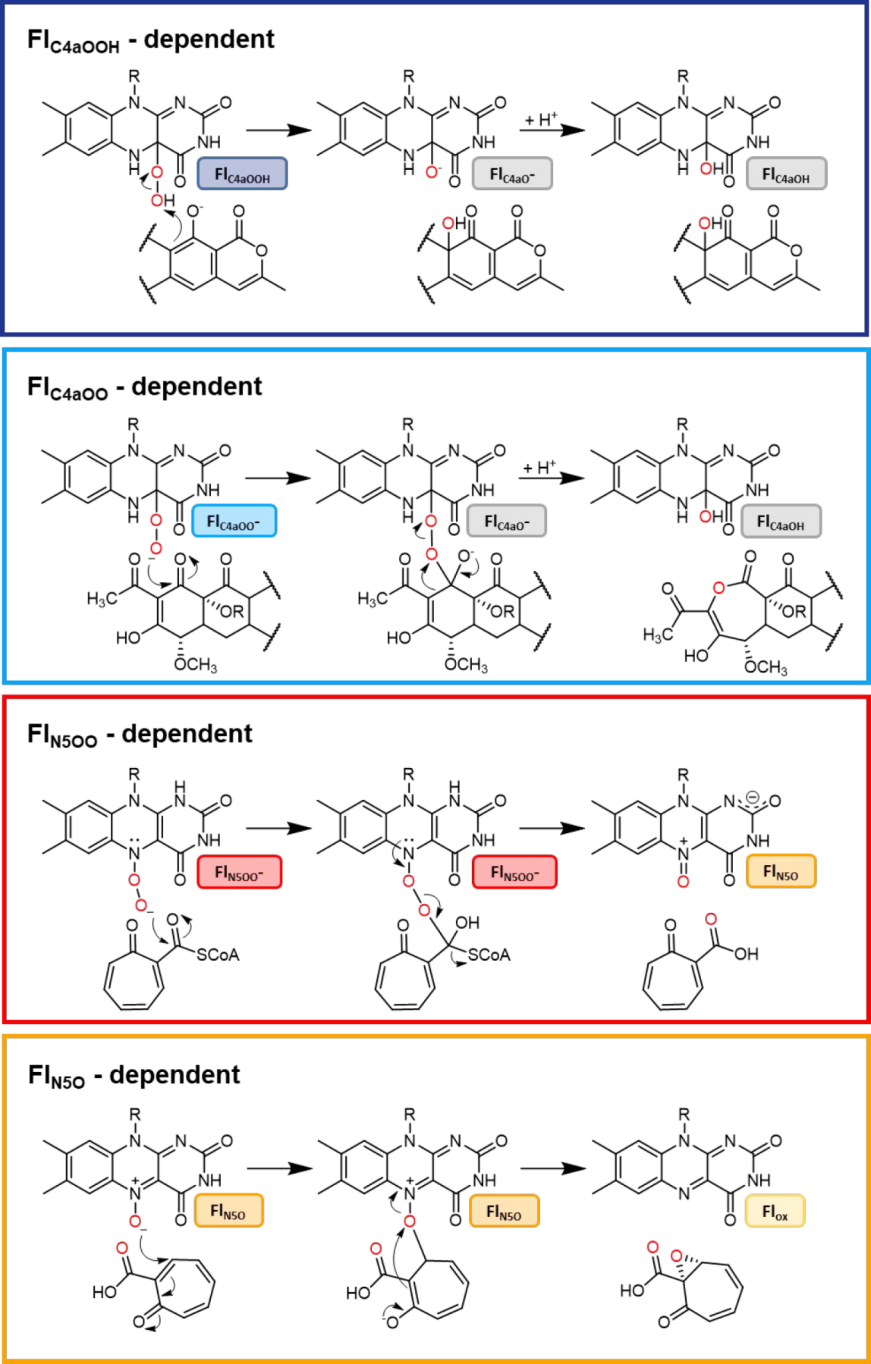
Reaction mechanisms of selected flavoprotein oxygenases involved in natural product biosynthetic pathways. Dark blue box, Fl_C4aOOH_-dependent aromatic hydroxylation of reduced collinone catalyzed by the group A FPMO GrhO5 (Uniprot entry Q8KSX7). Light blue box, Fl_C4aOO_-dependent monooxygenation of premithramycin B to the corresponding lactone catalyzed by the BVMO MtmOIV (Uniprot entry Q194P4). Red and orange boxes, suggested flavoprotein dioxygenase functionality of TdaE (Uniprot entry I7DWF3) presumably involving the consecutive Fl_N5OO_-dependent coenzyme A-ester oxygenolysis (red box) and Fl_N5O_-dependent epoxidation of the tropone-2-carboxylate intermediate (orange box). Introduced oxygen atoms derived from O_2_ are colored red. All shown flavin-oxygen adducts are formed from the reaction of Fl_red_ with O_2_.

Flavoprotein monooxygenases (FPMOs) have been studied for many decades and generally require the reduction of oxidized flavin (Fl_ox_) to Fl_red_ by external [e.g., NAD(P)H] or internal (substrate) electron donors prior to the reaction with O_2_ and the formation of covalent flavin-oxygen adducts. Typically, flavin-C4a-(hydro)peroxy [Fl_C4aOO(H)_] species then mediate the oxygenation of organic substrates such as natural product precursors. The electrophilic Fl_C4aOOH_ species is often employed to hydroxylate activated aromatic compounds such as phenols (Figure 2, dark blue box), while deprotonated, nucleophilic Fl_C4aOO_ species are used by Baeyer-Villiger monooxygenases (BVMOs) that typically convert ketones into lactones (Figure 2, light blue box). More rarely, N-hydroxylation reactions are being catalyzed by enzymes related to group B BVMOs, which will not be further discussed here.^13,14^ Both Fl_C4aOO_- and Fl_C4aOOH_-dependent catalytic mechanisms are commonly observed in the tailoring of natural products such as aromatic polyketides, as outlined below in more detail.

In 2013, however, EncM was the first enzyme shown to feature a stable flavin-N5-oxide (Fl_N5O_) in the resting state,^15^ which is presumably formed from a short-lived flavin-N5-peroxide (Fl_N5OO_) precursor and allows the hydroxylation of a reactive acyl-carrier protein-bound linear octaketide chain in enterocin (**1**) biosynthesis.^15−19^ N5-oxygenated flavins were soon shown to be a broader theme in flavin enzymology and reported in a range of group C FPMOs that catalyze unusual redox-neutral oxygenations of C–N, C–S, and C–Cl bonds, thereby resembling hydrolyses, as part of catabolic pathways in bacteria.^20−22^ While the exact oxygen transfer and catalytic mechanisms require further investigation, recent studies suggested the involvement of Fl_N5OO_ in effectuating these “pseudo-hydrolyses” of carbon–heteroatom bonds via formal transfer of an [OH]^−^ from the Fl_N5OO_ species.^6,23^ Remarkably, just lately the flavoenzyme TdaE from bacterial tropone natural product biosynthesis was shown to surprisingly function as an internal flavoprotein dioxygenase that may employ Fl_N5OO_ and Fl_N5O_ species for the consecutive CoA–thioester bond oxygenolysis and tropone ring epoxidation, respectively (Figure 2, red and orange boxes).^24^ In the next paragraphs, we will illustrate in more detail how these oxygenating species are exploited to mediate diverse tailoring reactions during the maturation of bacterial natural products and highlight structural and mechanistic features of selected flavoenzymes.

## Flavoenzyme-Dependent Aromatic Polyketide Tailoring Reactions in Actinobacteria

**Figure 3:**
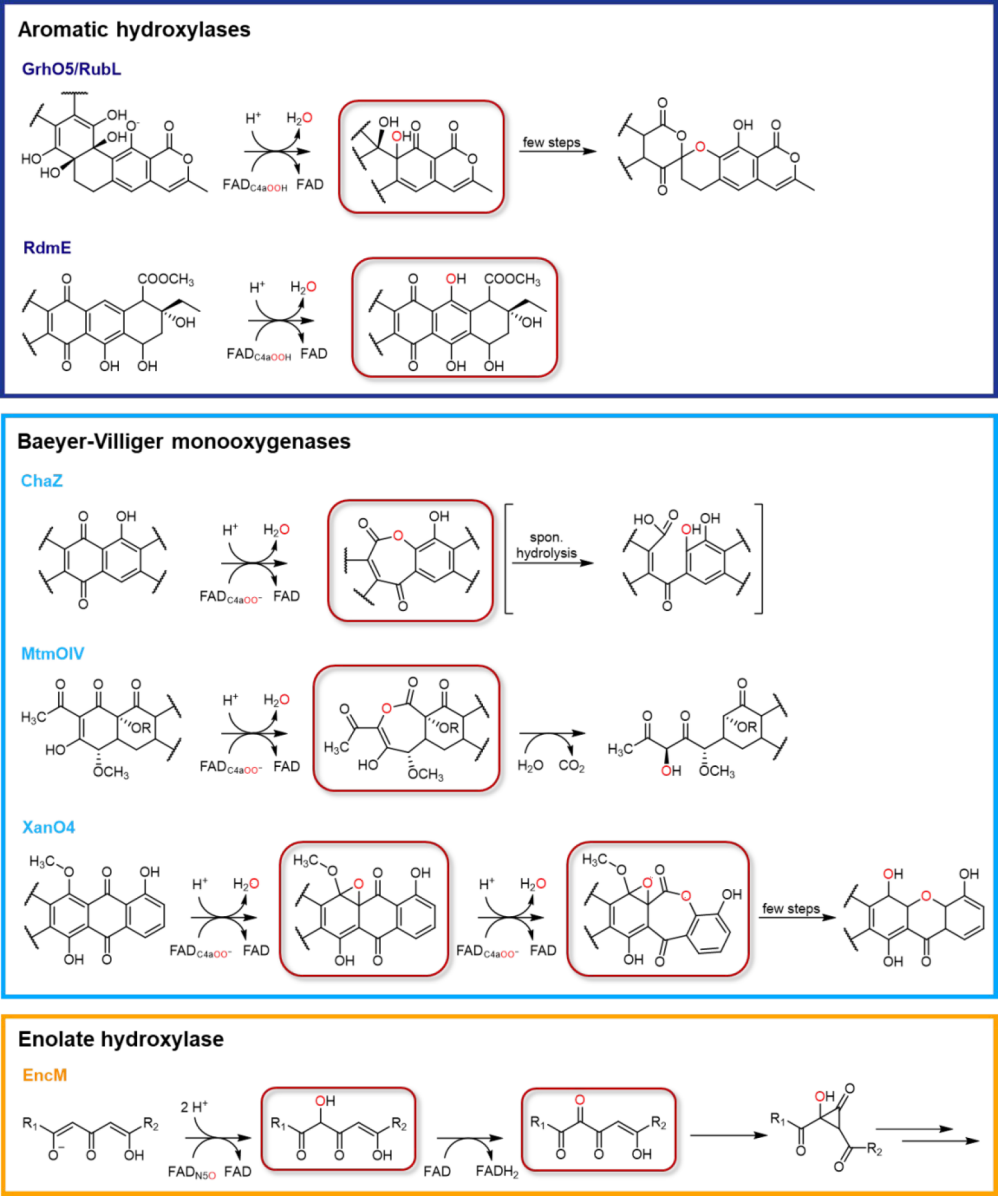
Selected bacterial flavoenzyme-dependent aromatic polyketide tailoring reactions. Dark blue box, reactions catalyzed by the aromatic hydroxylases (class A FPMOs) GrhO5 (Uniprot entry Q8KSX7) and RdmE (Uniprot entry Q54530) involved in the biosynthesis of griseorhodin A (**3**) and daunorubicin (**2**), respectively. Light blue box, Baeyer-Villiger-type monooxygenations proposed for MtmOIV (Uniprot entry Q194P4), XanO4 (Uniprot entry I1SKW8), and ChaZ (Uniprot entry Q4R0K8), participating in biosynthesis of mithramycin B (**7**), xantholipin, and chartreusin (**8**), respectively. These enzymes are assumed to rely on classical C4a-oxygenated flavins for catalysis. Orange box, redox tailoring reactions catalyzed by EncM (Uniprot entry Q9KHK2) in the biosynthesis of enterocin (**1**) depending on a flavin-N5-oxide as the oxygenating species. Aside from EncM, TdaE involved in bacterial tropone biosyntheses likely also utilizes N5-oxygenated flavins as catalytically active species (see Figure 2). Proposed products of the oxygenation reactions discussed in this article are highlighted with red boxes. Note that only selected steps are shown for each enzyme reaction.

Within the past 10–15 years, numerous flavoenzymes involved in (late) redox tailoring steps of aromatic polyketide biosynthetic pathways in Actinobacteria have been identified and biochemically as well as structurally characterized.^25^ It was found that many of these enzymes are group A FPMOs,^16,26−36^ catalyzing either aromatic hydroxylations (Figure 2, dark blue box) or BV monooxygenations (Figure 2, light blue box), which in some cases are followed by complex structural rearrangements that are controlled by the same enzymes and ultimately yield dedicated on-pathway intermediates. Aromatic hydroxylases, such as GrhO5^28^ and RdmE,^26,37^ typically depend on electrophilic FAD_C4aOOH_ as the oxygenating species to catalyze the *ortho* or *para* hydroxylation of phenolic compounds (or heterocyclic derivatives). RdmE was shown to mediate the oxygenation of aklavinone to directly yield the on-pathway intermediate ε-rhodomycinone en route to anthracyclines such as daunorubicin (**2**). Similarly, GrhO5 and its functional homologue RubL {from griseorhodin A (**3**) and rubromycin [e.g., β-rubromycin (**4**)] biosynthesis, respectively} were found to hydroxylate collinone (**5**) *ortho* to the phenolic hydroxyl group of ring E (Figure 3, dark blue box). However, in this case, introduction of the alcohol group additionally triggers two ring-cleaving retro-aldol reactions followed by carbonyl hydration, several tautomerizations, and an oxidation step to finally afford the [6,6]-spiroketal-containing compound dihydrolenticulone (**6**) (Figure 3, dark blue box).^28,29^

BV monooxygenases like ChaZ, MtmOIV, and XanO4 also require flavin C4a-oxygen adducts for catalysis; however, in contrast to the aromatic hydroxylases, the nucleophilic anionic form of the peroxide (FAD_C4aOO_) is used (Figure 3, light blue box). When the carbonyl moieties are nucleophilically attacked, lactone intermediates are generated, which are mostly unstable and tend to undergo spontaneous hydrolysis, often limiting direct mechanistic proof. Studies by Gibson et al.^35^ and Jiao et al.,^36^ nevertheless, clearly showed that both MtmOIV and ChaZ catalyze the formation of complex lactone-containing compounds from premithramycin B and resomycin C, respectively (Figure 3, light blue box). Even though premithramycin B-lactone was sufficiently stable to allow its direct detection by high-performance liquid chromatography analysis, *in vivo* this compound is short-lived and spontaneously hydrolyzes and decarboxylates to mithramycin DK, which is further converted to mithramycin (**7**) by MtmW.^34,35^ The lactone moiety in the resomycin C derivative, in contrast, is highly unstable, precluding its direct detection by chromatographic methods. However, it appears to be the true substrate of the downstream enzyme ChaE that together with additional enzymes ultimately forms chartreusin (**8**), once more underlining how well enzymes are primed for their specific tasks.^36^ In addition, XanO4^33^ and RslO9,^30^ which catalyze redox tailoring reactions in the biosynthesis of xantholipin and rishirilides [e.g., rishirilides A (**9**) and B], have been suggested to function as BV monooxygenases. In both cases, enzyme-mediated lactone formation is proposed to set off substantial structural rearrangements, leading to the formation of a xanthone-containing metabolite and rishirilides, respectively (Figure 3, light blue box). BVMO activity coupled to skeletal rearrangement was also implicated for the homologous FMN/FAD-dependent GilOII and JadG involved in gilvocarcin and jadomycin biosynthesis, which surprisingly resemble cofactor-free anthrone oxygenases rather than typical FPMOs.^38,39^

A remarkable exception from the prevailing FAD_C4aOO(H)_ paradigm in the redox tailoring of bacterial aromatic polyketides is found for EncM, which employs FAD_N5O_ as oxygenating species.^15,16,40^ The EncM-FAD_N5O_-catalyzed hydroxylation of an enolate moiety followed by the FAD_ox_-mediated oxidation of the newly introduced alcohol group affords a highly reactive 1,2,3-triketone motif as part of the polyketide chain (Figure 3, orange box). This compound is then suggested to spontaneously undergo a complex Favorskii-type rearrangement as well as ring-forming aldol condensations and heterocycle formation to end up with the characteristic **1** ring system in the form of desmethyl-5-deoxyenterocin. Strikingly, upon oxidation of the alcohol group, FAD_red_ is generated, which may react with O_2_ to afford the FAD_N5O_ species and is therefore primed for the next catalytic cycle. As such, EncM uses its substrate as an electron donor for flavin reduction without the need for external reductants like NAD(P)H. In contrast to other so-called internal oxygenases, however, EncM represents an inverted internal FPMO as it catalyzes the oxygenation prior to substrate dehydrogenation, which is enabled by the stable FAD_N5O_ species maintained in the resting state of EncM.^6,10,15,16,18^ EncM showcases how cofactors can be fine-tuned, as the high reactivity of the linear polyketide substrate is offset by an attenuated, less reactive oxygenating species. The usage of the stable FAD_N5O_ may be further advantageous due to the prevention of hydrogen peroxide formation by uncoupling, which is considered the undesired collapse of the FAD_C4aOO(H)_ species in classical FPMO catalysis.

## Flavoenzyme-Dependent Tropone Biosynthesis in Proteobacteria

Tropone natural products such as tropodithietic acid [TDA (**10**)], roseobacticides [e.g., roseobacticide A (**11**)], ditropolonyl sulfide (**12**), and tropolone (**13**) are known for their antimicrobial, antifungal, and anticancer activities as well as for their role as signaling molecules (e.g., in quorum sensing).^3,41−47^ All of these compounds contain a seven-membered aromatic carbon ring system, decorated with a keto function contributing to the aromaticity of these molecules.^48^ Isotope labeling experiments combined with gene knockout studies have shown that bacterial tropone natural products are predominantly derived from phenylacetic acid (**14**)^49−52^ and that sulfur amino acid and glutathione metabolism are crucial for sulfur incorporation.^53,54^ In the past decade, the investigation of **14** catabolism in bacterial species^55−60^ led to the serendipitous discovery of the shunt product 2-hydroxycyclohepta-1,4,6-triene-1-formyl-CoA (**15**) that because of its structural characteristics was proposed as the universal precursor for tropone natural products in bacteria.^61^

Only recently an acyl-CoA dehydrogenase (ACAD)-like enzyme (TdaE), originally identified in the *tda* biosynthetic gene clusters (BGCs) of marine *Roseobacter* (e.g., *Phaeobacter inhibens*)^52,62^ and recently in the putative tropone natural product BGCs of *Burkholderia plantarii*, *Burkholderia cenocepacia*, and others,^24^ was characterized for the first time and shown to accept this universal precursor as a substrate.^24^ Strikingly, this enzyme not only oxidizes **15** to the ketone derivative, as expected from its annotation as ACAD, but also oxygenolytically cleaves the CoA ester to yield the corresponding carboxylic acid before introducing an epoxide functionality into the tropone backbone, thereby affording (2*R*,3*R*)-2,3-epoxytropone-2-carboxylate (**16**). The FAD-dependent TdaE uses the reducing equivalents from the initial oxidation reaction to generate FAD_red_, which then reacts with O_2_ most probably to form FAD_N5OO_. The unique chemical properties of the FAD_N5OO_ subsequently allow for the redox-neutral cleavage of the thioester bond, yielding tropone-2-carboxylate and FAD_N5O_ (Figure 2, red box). The FAD_N5O_ is then proposed to attack the C8 position of the tropone ring to trigger regio- and stereospecific epoxide formation and the regeneration of FAD_ox_ for another catalytic cycle (Figure 2, orange box). Notably, the currently proposed TdaE mechanism involves two consecutive oxygen transfer reactions similar to some previously reported FPMOs that catalyze sequential monooxygenations. In contrast to these enzymes, however, TdaE achieves this with only one substrate-derived reducing equivalent [no external reducing agents such as NAD(P)H are needed] and presumably by transferring both oxygen atoms from the same molecule of O_2_. Hence, TdaE represents a remarkably efficient enzyme and can be considered the first internal flavoprotein dioxygenase.^24^ The TdaE product then most likely serves as an advanced precursor for tropolone (**13**) or the sulfur-containing tropodithietic acid (**10**), roseobacticides (e.g., **11**), and ditropolonyl sulfide (**12**) in various bacteria. For example, **16** spontaneously decarboxylates to **13**, which functions as a virulence factor in a rice seedling disease caused by *B. plantarii*, while the same compound is likely processed in other bacteria by sulfur-incorporating enzymes to ultimately yield, e.g., **10**, **11**, or **12**.^24^ It is still unclear how sulfur incorporation exactly proceeds, although the chemical properties of **16** including the reactive epoxide moiety seem to be well suited for nucleophilic sulfur incorporation. In accordance with this central functionality for tropone biosynthesis in bacteria, bioinformatic studies revealed TdaE homologues in a variety of α-, β-, and γ-proteobacteria, of which several are known producers of either tropolone (derivatives) or TDA.^63−66^ However, *Paracoccus* sp. as well as *Pseudomonas* sp., *Pseudoduganella* sp., and *Paraburkholderia* sp. also encode TdaE homologues and may produce (potentially novel) tropone natural products.^24^

## Challenges for the Prediction of Unusual Flavoenzyme Functionalities and Their Exploitation in Natural Product Bioengineering

One of the most exigent challenges in natural product biosynthesis is the prediction of detailed tailoring enzyme functionalities based on sequence homology. For bacterial aromatic polyketides, this especially applies to compounds with a framework that undergoes extensive modifications and rearrangements. If the corresponding BGCs encode a manageable amount of putative tailoring enzymes, canonical reactions (e.g., ketoreduction or methylation) are often unproblematic to assign to enzyme candidates in contrast to the often unique skeletal rearrangements that give rise to the perplexing structural complexity of many natural products. Typically, such backbone modifications are triggered by redox reactions, and flavoenzymes hereby clearly adopt the most prominent role in bacterial aromatic polyketide biosynthesis. Likely, this results from the chemical properties of the encountered biosynthetic intermediates that well match the reactivities of the accessible FPMO oxygenating species; i.e., activated (functionalized) aromatic rings and ketones that are often present in cyclized polyketides are prone to react with typical organic peroxides such as the Fl_C4aOO^–^__and_ Fl_C4aOOH_ species, while the FAD_N5O_ appears to be adequate for the much more reactive linear polyketide chain.

A main issue for the prediction efforts is the often ostensible lack of structural motives that could be associated with certain enzyme functionalities. For example, BVMOs normally feature dedicated catalytic bases to deprotonate the Fl_C4aOOH_ species (exemplary p*K*_a_ values are 8.4 for the BVMO cyclohexanone monooxygenase and >10 for aromatic hydroxylases^67−69^). In contrast, group A members that catalyze BV monooxygenations [known as “type III” or odd-type (O) BVMOs] lack obvious bases. However, local p*K*_a_ values may be controlled by more complex interactions with the substrate and multiple active site residues. This is exemplified by another type of flavoenzyme, vanillyl alcohol oxidases (VAOs), which tightly interact with the phenolic hydroxyl group of their substrates via several amino acid side chains. This results in a decrease in the p*K*_a_ by ∼2 units and thus in substrate activation.^70^ Alternatively or in addition to that, the precise positioning of the substrate with respect to the oxygenating species in the active site of FPMOs could largely determine the nature of the oxygenation reaction.

We surmise that flavin-dependent redox tailoring enzymes that mediate skeletal rearrangements subsequent to oxygen transfer may mostly provide protected reaction chambers that are conducive to the desired reactions while precluding alternative routes and thus shunt product formation via “negative catalysis”.^71,72^ For instance, many of the flavoenzymes described herein adopt seemingly canonical folds with inconspicuous active sites, as exemplified by the spiroketal synthases GrhO5 and RubL.^28^ The **3** BGC, e.g., encodes additional predicted group A FPMOs that are highly similar to GrhO5, i.e., GrhO8 (45.5% amino acid identity, 98% coverage) and GrhO9 (44% amino acid identity, 94% coverage), which most likely catalyze conventional aromatic hydroxylations in preceding tailoring steps.^29,73^ Recent structural and biochemical investigation of GrhO5/RubL revealed many classical characteristics of the mechanistically complex group A FPMOs. In addition, a cluster of basic amino acid side chains proved to be crucial for product formation, presumably not only by binding and activating **5** for aromatic hydroxylation but also by promoting formation of the anionic intermediates for the subsequent backbone rearrangement en route to **6**.^28^ However, no candidate for a catalytic amino acid required for these reactions could be identified, implying that the reaction cascade might be primarily driven by the high innate energy of hydroxylated collinone. A similar scenario can be found for EncM, which is a member of the VAO/PCMH flavoprotein family^74,75^ that typically comprises dehydrogenases and oxidases rather than oxygenases and has been tentatively proposed as the first member of group H FPMOs.^6^ EncM also lacks evident catalytic amino acid residues for the Favorskii rearrangement and the cyclization reactions. Instead, an elongated L-shaped substrate binding tunnel separates the reactive ketones and enol(ate) groups of the linear polyketide chain from each other, thereby counteracting spontaneous undesirable cyclization and aromatization, while promoting the FAD_N5O_-mediated hydroxylation.^11,15,18^ However, the catalysis of skeletal rearrangements by such enzymes cannot be ruled out, and it is even conceivable that the flavin cofactors partake as chassis in some of these reactions. Similar to the seemingly unpredictable roles of GrhO5 or EncM in aromatic polyketide biosynthesis, the ACAD-like TdaE was found to exhibit surprising dioxygenase activity in the tailoring of tropone natural products.^24^ While FPMOs with an ACAD fold normally belong to group D FPMOs,^7^ TdaE is more similar to classical ACADs based on homology modeling (even though its active site residues are likely distinct) and does not closely resemble any previously characterized FPMO.^24^ These findings underscore the difficulties in predicting flavoenzyme catalysis based on classical approaches (e.g., BLAST searches, sequence alignments, and homology modeling).

It is a tantalizing idea to employ “talented” tailoring enzymes for the production of natural product derivatives via biotechnological approaches *in vitro* or *in vivo*, e.g., by broadening the substrate scope. However, there are significant hurdles that impede such endeavors; e.g., group A FPMOs such as GrhO5 or RslO9 feature complex catalytic cycles and typically only react with NAD(P)H in the presence of their native substrate (see refs (6−8), (76), and (77) for further information). It is currently unclear how relaxed the substrate specificity for such enzymes is and if these proofreading mechanisms can be bypassed by maintaining certain substrate features. Moreover, protein dynamics required for catalysis (often overlooked by X-ray crystallography) or active site constrictions might thwart such efforts; e.g., EncM’s distinctive substrate binding tunnel might be unsuitable for more bulky substrate analogues.^15^ Aside from enzyme characteristics, the procurement of substrate (analogues) may also pose substantial challenges due to high reactivity and instability. The rational design of flavoenzymes, e.g., with the aim of broadening the substrate scope, also often remains a “trial and error” approach because general rules about how amino acid replacements affect overall protein stability and cofactor functionalization are lacking. For example, subtle changes in the vicinity of the flavin cofactors may result in unforeseen effects with respect to formation of the different oxygenating species that might be mostly controlled by the approach of O_2_ to Fl_red_ as well as the protonation state of the transiently formed flavin semiquinone radical en route to C4a or N5-oxygenated flavins.^6^ Nonetheless, successful production of natural product analogues using FPMOs is feasible, e.g., by exploiting the relaxed substrate specificity of EncM, which allowed the generation of rearranged enterocin derivatives with modified/substituted terminal benzene ring,^78^ or by rational engineering of the two-component FPMO HpaBC to extend its substrate scope.^79,80^

To date, it has been assumed that most FPMOs employ the pervasive Fl_C4aOO(H)_ species for catalysis. However, often direct evidence is missing and typically can be obtained only by sophisticated stopped-flow spectroscopy and pre-steady state kinetics. Consequently, many enzymes relying on N5-oxygenated flavins may have been overlooked so far. Notably, stopped-flow spectroscopy so far has failed to provide evidence for the presumably very short-lived Fl_N5OO_ species,^15,16,23,81^ whereas the stable Fl_N5O_ can be identified by ultraviolet–visible spectroscopy (even though its spectrum is deceivingly similar to that of Fl_ox_) or mass spectrometry despite the fact that it is susceptible to reduction to Fl_ox_, which may impede its detection.^18^ Finally, the reactivities in particular of the Fl_N5OO_ and Fl_N5O_ species remain poorly explored due to the small number of reported enzymes. So far, the FAD_N5O_ has been shown to mediate enolate hydroxylation^15^ and presumably tropone epoxidation^24^ conceivably involving ionic or radical mechanisms, while the FAD_N5OO_ appears to primarily effectuate carbon–heteroatom bond cleavage reactions via redox-neutral “pseudo-hydrolyses”,^6,23^ but possibly also by more conventional oxygen transfers.^81^
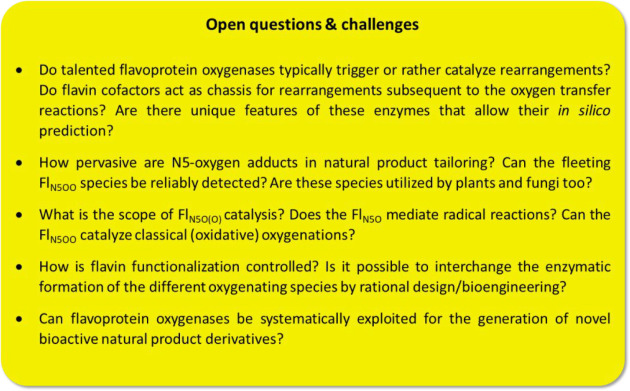


## Summary and Outlook

In this Perspective, we briefly highlighted recent developments and challenges in the field of flavoenzyme-mediated redox tailoring of bacterial natural products. As plants and fungi also make use of flavoenzymes in secondary metabolism,^82^ it would not come as a surprise if similar catalytic mechanisms would be reported in the future, e.g., involving FAD_N5O(O)_ adducts, possibly even in the biosynthesis of aromatic polyketides or tropones that are also generated by these organisms. The identification of “talented” tailoring enzymes in biosynthetic pathways is often challenging, and their application is not straightforward, also because many of the complex skeletal rearrangements may be driven forward by the high energy of key intermediates rather than being catalyzed. This means that the involved flavoenzymes might merely provide a “starting shot” for the ensuing cascade-like reactions in the form of canonical hydroxylations or BV monooxygenations, while precluding unwanted side reactions. It thus will be interesting to see the extent to which these enzymes can be exploited in the future for the generation of bioactive natural product derivatives.
